# Caffeine modulates glucocorticoid-induced expression of CTGF in lung epithelial cells and fibroblasts

**DOI:** 10.1186/s12931-017-0535-8

**Published:** 2017-03-23

**Authors:** Markus Fehrholz, Kirsten Glaser, Christian P. Speer, Silvia Seidenspinner, Barbara Ottensmeier, Steffen Kunzmann

**Affiliations:** 1 University Children’s Hospital, University of Wuerzburg, Josef-Schneider-Str. 2, 97080 Wuerzburg, Germany; 2 Clinic of Neonatology, Buergerhospital Frankfurt am Main, Nibelungenallee 37-41, 60318 Frankfurt am Main, Germany

**Keywords:** Airway remodeling, Bronchopulmonary dysplasia, Caffeine, CCN2, CTGF, Fibrosis, Glucocorticoids, H441, IMR-90

## Abstract

**Background:**

Although caffeine and glucocorticoids are frequently used to treat chronic lung disease in preterm neonates, potential interactions are largely unknown. While anti-inflammatory effects of glucocorticoids are well defined, their impact on airway remodeling is less characterized. Caffeine has been ascribed to positive effects on airway inflammation as well as remodeling. Connective tissue growth factor (CTGF, CCN2) plays a key role in airway remodeling and has been implicated in the pathogenesis of chronic lung diseases such as bronchopulmonary dysplasia (BPD) in preterm infants. The current study addressed the impact of glucocorticoids on the regulation of CTGF in the presence of caffeine using human lung epithelial and fibroblast cells.

**Methods:**

The human airway epithelial cell line H441 and the fetal lung fibroblast strain IMR-90 were exposed to different glucocorticoids (dexamethasone, budesonide, betamethasone, prednisolone, hydrocortisone) and caffeine. mRNA and protein expression of CTGF, TGF-β1-3, and TNF-α were determined by means of quantitative real-time PCR and immunoblotting. H441 cells were additionally treated with cAMP, the adenylyl cyclase activator forskolin, and the selective phosphodiesterase (PDE)-4 inhibitor cilomilast to mimic caffeine-mediated PDE inhibition.

**Results:**

Treatment with different glucocorticoids (1 μM) significantly increased CTGF mRNA levels in H441 (*p* < 0.0001) and IMR-90 cells (*p* < 0.01). Upon simultaneous exposure to caffeine (10 mM), both glucocorticoid-induced mRNA and protein expression were significantly reduced in IMR-90 cells (*p* < 0.0001). Of note, 24 h exposure to caffeine alone significantly suppressed basal expression of CTGF mRNA and protein in IMR-90 cells. Caffeine-induced reduction of CTGF mRNA expression seemed to be independent of cAMP levels, adenylyl cyclase activation, or PDE-4 inhibition. While dexamethasone or caffeine treatment did not affect TGF-β1 mRNA in H441 cells, increased expression of TGF-β2 and TGF-β3 mRNA was detected upon exposure to dexamethasone or dexamethasone and caffeine, respectively. Moreover, caffeine increased TNF-α mRNA in H441 cells (6.5 ± 2.2-fold, *p* < 0.05) which has been described as potent inhibitor of CTGF expression.

**Conclusions:**

In addition to well-known anti-inflammatory features, glucocorticoids may have adverse effects on long-term remodeling by TGF-β1-independent induction of CTGF in lung cells. Simultaneous treatment with caffeine may attenuate glucocorticoid-induced expression of CTGF, thereby promoting restoration of lung homeostasis.

## Background

Bronchopulmonary dysplasia (BPD) still represents a major morbidity of preterm birth [1]. It has been deemed an evolving process of chronic lung inflammation and lung injury. Besides structural immaturity, pre- and postnatal inflammation has been considered a principle mechanism in the initiation and aggravation of BPD. Various adverse conditions, such as mechanical ventilation, may amplify the inflammatory response and contribute to severe lung injury [2–9]. The latter is characterized by impaired alveolarization and impaired vascular development and culminates in severe airway remodeling with interstitial and vascular fibrosis [10–13].

Connective tissue growth factor (CTGF), also known as CCN family protein 2 (CCN2), is a matricellular protein, that plays a key role in tissue development and remodeling, interacting with a variety of other growth factors, such as transforming growth factor (TGF)-β [14]. It has been deemed a critical role in the pathogenesis of various forms of adult pulmonary fibrosis and vascular disease [15, 16]. Both growth factors have been acknowledged as central mediators promoting and accelerating fibrosis as well as pathological airway remodeling [12, 17, 18]. In pulmonary fibrosis, CTGF seems to be predominantly localized to proliferating alveolar type II (ATII) cells and activated fibroblasts [19] and, thus, may play a central part as pro-fibrotic mediator. In the neonatal lung, increased expression of CTGF seems to be induced by mechanical ventilation and hyperoxia, suggesting that CTGF may contribute to the pathogenesis of BPD [20–22]. In addition, in neonatal mice, a conditional overexpression of CTGF in ATII cells was shown to induce lung fibrosis, resulting in a BPD-like architecture [10]. These data may underline a key role of CTGF in tissue fibrosis and airway remodeling, both displaying important features of BPD. However, underlying mechanisms of the transcriptional modulation of CTGF, considered to be its predominant form of regulation [23], may be complex and might depend on the particular disease or the affected organ [24]. While TGF-β seems to induce CTGF gene expression [23], tumor necrosis factor alpha (TNF-α), among other factors, has been shown to reduce expression of CTGF [25].

Besides, there is considerable evidence of an even more complex interplay of CTGF and TGF-β [26]. CTGF seems to enhance the impact of TGF-β in the context of pro-inflammation [27]. It may act as a co-factor for TGF-β, but can also activate TGF-β in extracellular matrix signaling [28]. In pro-inflammatory lung injury, in concert with TGF-β, CTGF seems to trigger the production of remodeling molecules in the extracellular matrix [27]. Increased expression of both TGF-β1 and CTGF has been associated with severe forms of BPD [6, 22, 29–32].

In preterm infants, the administration of glucocorticoids aiming at the attenuation of BPD has long been subject to controversy [33, 34]. Glucocorticoids may be used to accelerate weaning from respiratory support [35] and to treat or prevent chronic inflammatory diseases [36] as well as fibrotic lung disease [12]. However, potential adverse effects on long-term airway remodeling are a matter of ongoing debate [37, 38]. Effects of glucocorticoid administration on CTGF signaling, in particular, have not been sufficiently investigated, so far. Potential adverse effects on the lung epithelium demand further studies on the impact of glucocorticoids on airway remodeling [12].

The methylxanthine caffeine is commonly used to reduce apnea of prematurity [39, 40]. Of note, caffeine treatment has been associated with reduced incidences of BPD [41] and the prevention of hyperoxia-mediated pulmonary inflammation and lung injury [42, 43]. Although caffeine has been demonstrated anti-inflammatory [44] and antifibrotic effects [45, 46], its potential impact on airway remodeling has not been investigated in detail.

We recently demonstrated that caffeine is able to antagonize TGF-β1 induced upregulation of CTGF on the transcriptional and translational level [47] and that gene expression-related additive and synergistic effects exist for caffeine in combination with dexamethasone [48, 49]. At higher concentrations, caffeine may act as an unspecific inhibitor of PDEs increasing intracellular levels of cAMP. At lower concentrations, predominantly nonselective antagonism on adenosine receptors but also roles in histone acetylation and deacetylation have been reported [50–53].

It is of high relevance to better characterize potential pro-fibrotic effects of glucocorticoids and caffeine in the context of BPD and airway remodeling. Considering the importance of CTGF for normal lung development and pro-fibrotic processes independent of the underlying disease [14], thrown off regulatory balance in the preterm lung during BPD, a modulation of CTGF expression might be vitally important to counteract restricted lung development caused by fibrotic processes and pathologic airway remodeling. The current study addressed the impact of glucocorticoids and caffeine, alone or in combination, on CTGF expression in different lung cells.

## Methods

### Reagents

Caffeine, dexamethasone, budesonide, betamethasone, prednisolone, hydrocortisone, 8-Br-cyclic adenosine monophosphate (cAMP), forskolin, cilomilast, and recombinant human TNF-α were purchased from Sigma-Aldrich (St. Louis, CA).

### Cells

Human airway epithelial cells NCI-H441 (H441) and the fetal lung fibroblast strain IMR-90 were purchased from ATCC (LGC Standards, Teddington, UK) and cultured as described, respectively, without sodium pyruvate and nonessential amino acids in case for IMR-90 [48, 54]. Incubation was carried out at 37 °C in a humidified atmosphere with 5% CO_2_. For stimulation assays with glucocorticoids and caffeine, H441 and IMR-90 cells were seeded on six well plates (Greiner, Frickenhausen, Germany) until 80% confluence was reached and subsequently incubated with substances in growth medium as indicated in a total volume of 1 mL until further processing. Preliminary dose–response experiments using concentrations of 100 μM, 1 mM, and 10 mM caffeine revealed highest effects for 10 mM. Therefore the latter concentration was used throughout all experiments.

### Neutralization assay

To neutralize extracellular TNF-α, antibodies against human TNF-α (clone 2C8; Abcam, Cambridge, United Kingdom) were used in a concentration of 5 μg/mL.

### RNA extraction and RT-PCR

For RNA extraction, cells were treated as indicated and total RNA was isolated using NucleoSpin® RNA Kit (Macherey-Nagel, Dueren, Germany) according to the manufacturer’s protocol. For quantification of total RNA, a Qubit® 2.0 Fluorometer (Thermo Fisher Scientific, Waltham, MA) was used as recommended by the manufacturer. Total RNA was eluted in 60 μL nuclease-free H_2_O (Sigma-Aldrich) and stored at −80 °C until reverse transcription. For RT-PCR, 1 μg of total RNA was reverse transcribed using High Capacity cDNA Reverse Transcription Kit (Thermo Fisher Scientific) according to the manufacturer’s instructions. First strand cDNA was diluted 1 to 10 with deionized, nuclease-free H_2_O (Sigma-Aldrich) and stored at −20 °C upon analysis.

### Quantitative real time RT-PCR (qPCR)

For quantitative detection of mRNA, 10 μL of diluted first strand cDNA were analyzed in duplicates of 25 μL reactions using 12.5 μL iTaq™ Universal SYBR® Green Supermix (Bio-Rad Laboratories, Hercules, CA), 0.5 μL deionized H_2_O, and 1 μL of a 10 μM solution of forward and reverse primers (Sigma-Aldrich) as indicated in table 1. PCRs were performed on an Applied Biosystems® 7500 Real-Time PCR System (Thermo Fisher Scientific) using a 2-step PCR protocol after an initial denaturation at 95 °C for 10 min with 40 cycles of 95 °C for 15 s and 60 °C for 1 min. A melt curve analysis was performed at the end of every run to verify single PCR products. Levels of mRNAs were normalized to those of glyceraldehyde-3-phosphate dehydrogenase (GAPDH). Mean fold changes in mRNA expression were calculated by the ΔΔC_T_ method by Livak and Schmittgen [55].

**Table 1:** Primers for qPCR

Gene symbol	Sequence accession #	Orientation	Sequence [5′ to 3′]	Amplicon length [bp]
CTGF	NM_001901.2	forward	ACCCAACTATGATTAGAGCC	189
		reverse	TTGCCCTTCTTAATGTTCTC	
GAPDH	NM_002046.5	forward	CCATGGAGAAGGCTGGGG	195
		reverse	CAAAGTTGTCATGGATGACC	
TGFB1	NM_000660.5	forward	AATTCCTGGCGATACCTC	192
		reverse	TAGTGAACCCGTTGATGTC	
TGFB2	NM_001135599.2	forward	AGATTTGCAGGTATTGATGG	106
		reverse	ATTAGCAGGAGATGTGGG	
TGFB3	NM_003239.3	forward	CAAAGGCGTGGACAATGAG	200
		reverse	ACACAGCAGTTCTCCTCC	
TNF	NM_000594.3	forward	CAGCCTCTTCTCCTTCCT	188
		reverse	GGGTTTGCTACAACATGG	

### Immunoblotting

Immunoblotting was performed as previously described [48]. Blots were probed with primary antibodies to CTGF (clone L-20; Santa Cruz Biotechnology Inc., Santa Cruz, CA), and β-actin (926–42212; LI-COR, Lincoln, NE), followed by staining with corresponding IRDye® secondary antibodies (LI-COR) for 1 h at RT. Specific protein bands were visualized using an ODYSSEY® Infrared Imaging System (LI-COR). Accumulated signals were quantified using Image Studio Lite v5.0.21 (LI-COR).

### Statistical analysis

Results are given as means ± SD. Unless stated otherwise, data were analyzed using one way ANOVA with Bonferroni’s multiple comparison post hoc test. A *p*-value ≤ 0.05 was considered significant. All statistical analyses were performed using Prism® version 6 (GraphPad Software, San Diego, CA).

## Results

### Effect of glucocorticoids and caffeine on CTGF mRNA expression in H441 and IMR-90 cells

**Fig. 1:**
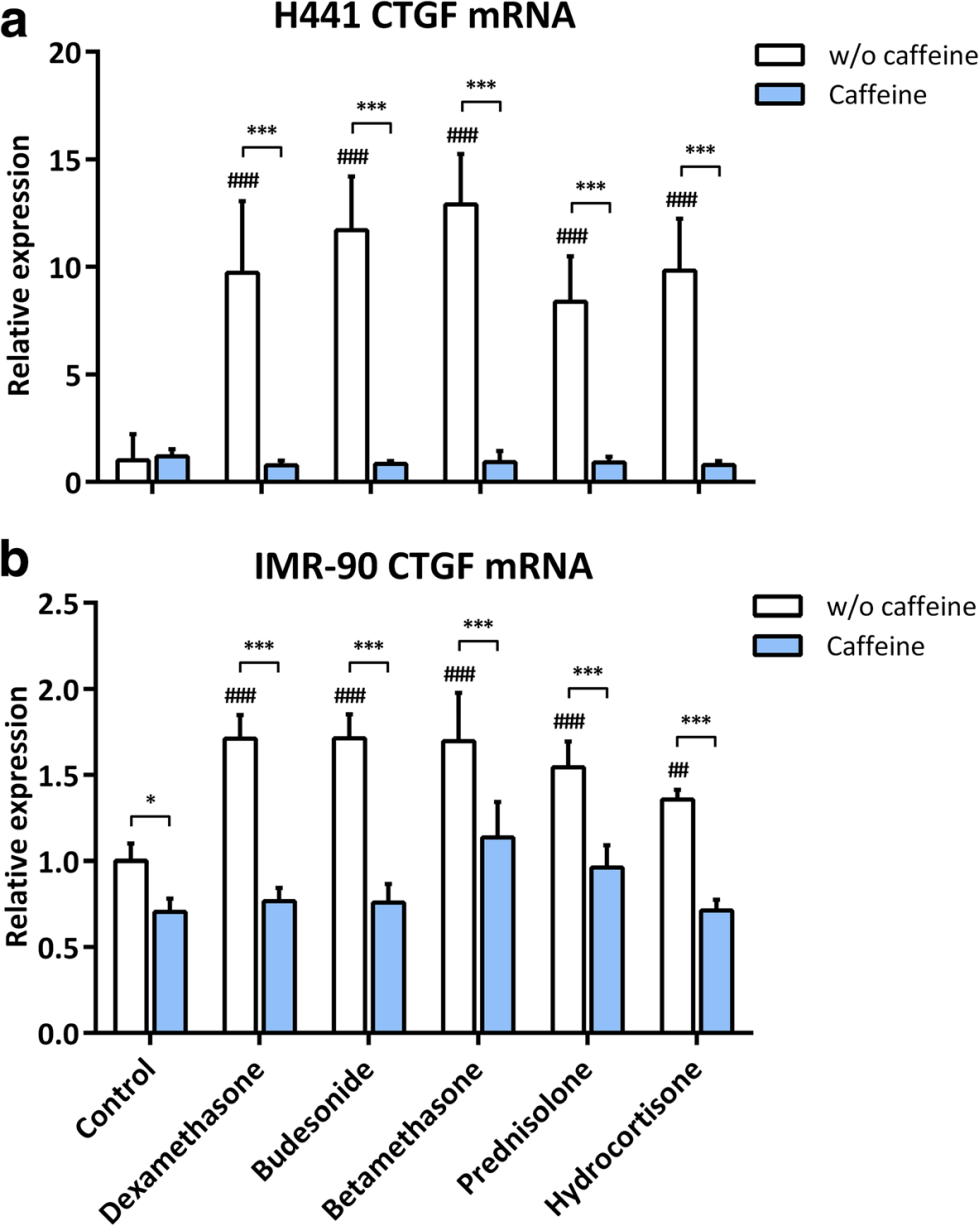
Reduction of glucocorticoid-induced CTGF mRNA expression in H441 and IMR-90 cells by caffeine. H441 and IMR-90 cells were treated with 1 μM of the indicated glucocorticoids and/or 10 mM caffeine for 24 h. qPCR against CTGF mRNA was performed as described in Methods. CTGF mRNA levels of H441 cells (**a**) and IMR-90 cells (**b**) were normalized to GAPDH, and fold differences compared to untreated cells were calculated. Means + SD of n ≥ 3 independent experiments are shown. ## *p* < 0.01, and ### *p* < 0.001 compared to untreated controls; *p < 0.05 and ***p < 0.001 compared to cells treated with the corresponding glucocorticoid

To investigate a potential impact of glucocorticoids and/or caffeine on expression of CTGF mRNA, we treated H441 and IMR-90 cells with various glucocorticoids alone or in combination with caffeine. We observed a significant induction of CTGF mRNA for dexamethasone, budesonide, betamethasone, prednisolone, and hydrocortisone in H441 and IMR-90 cells. Highest levels of CTGF mRNA induction were reached in H441 cells (8.4 to 12.9-fold, *p* < 0.0001 for all glucocorticoids; Fig. 1a) followed by those in IMR-90 cells (1.4 to 1.7-fold, *p* = 0.0015 for hydrocortisone and *p* < 0.0001 for all other glucocorticoids; Fig. 1b). In contrast, exposure of both cell lines to caffeine did not induce CTGF mRNA expression, but significantly reduced basal CTGF mRNA levels in IMR-90 cells (*p* = 0.0221; Fig. 1b). For H441 cells, these effects were not statistically significant (Fig. 1a). Of note, simultaneous exposure of lung cells to glucocorticoids and caffeine significantly prevented glucocorticoid-induced CTGF expression in H441 as well as in IMR-90 cells (*p* < 0.0001 for all glucocorticoids in combination with caffeine; Figs. 1a and b).

### Effect of glucocorticoids and caffeine on CTGF protein expression in IMR-90 cells

**Fig. 2:**
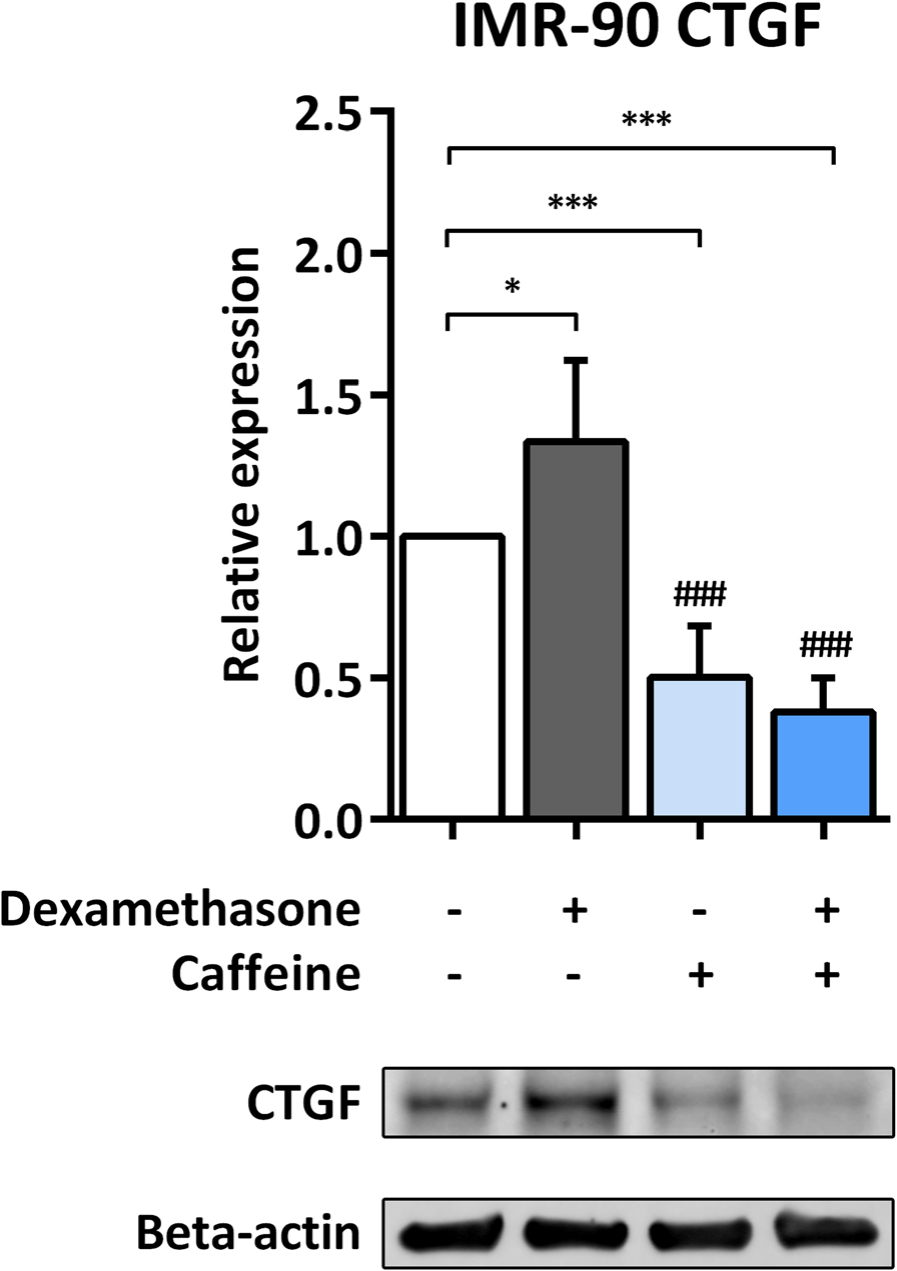
Treatment with caffeine reduces basal and dexamethasone-induced CTGF protein expression in IMR-90 cells. IMR-90 cells were treated with 1 μM dexamethasone and/or 10 mM caffeine for 24 h. Immunoblotting against CTGF and β-actin was performed as described in Methods. CTGF levels were normalized to those of β-actin, and fold differences compared to untreated cells were calculated. Means + SD of *n* = 4 independent experiments are shown. DEX, dexamethasone; *p < 0.05 and ***p < 0.001 compared to untreated controls; ### *p* < 0.001 compared to cells treated with dexamethasone

As far as protein expression was concerned, CTGF was undetectable in H441 cells for every condition at 24 h incubation (data not shown). In IMR-90 cells, a significant induction of CTGF was detected after a 24 h treatment with dexamethasone (1.3 ± 0.3-fold, *p* = 0.0289; Fig. 2). CTGF protein levels were significantly lower in IMR-90 cells treated with caffeine alone or in combination with dexamethasone in comparison to untreated cells (*p* = 0.009 and *p* = 0.0001, respectively) as well as in comparison to cells treated with dexamethasone alone (*p* < 0.0001 for both conditions; Fig. 2).

The given results indicate that CTGF expression is induced by glucocorticoids on a transcriptional and translational level in lung epithelial and fibroblast cells and that this induction may be prevented by simultaneous exposure to caffeine.

### Impact of dexamethasone and caffeine on TGF-β1-3 mRNA expression in H441 cells

**Fig. 3:**
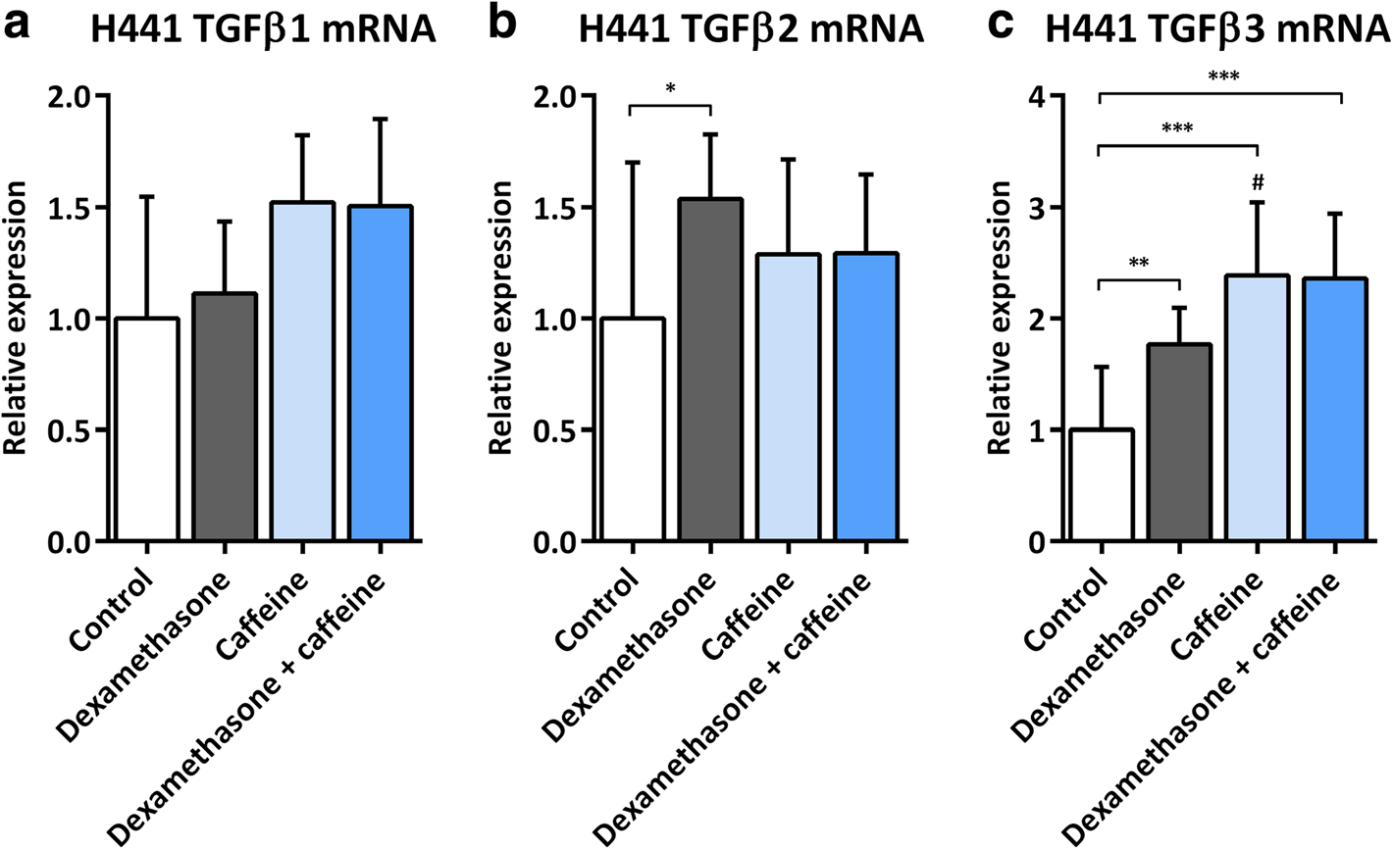
Impact of dexamethasone and caffeine on TGF-β1-3 mRNA expression in H441 cells. H441 cells were treated with 1 μM dexamethasone and/or 10 mM caffeine for 24 h. qPCR against TGF-β1-3 mRNA was performed as described in Methods. TGF-β1 (**a**), TGF-β2 (**b**), and TGF-β3 (**c**) mRNA levels of H441 cells were normalized to GAPDH, and fold differences compared to untreated cells were calculated. Means + SD of n ≥ 3 independent experiments are shown. *p < 0.05, **p < 0.01, and ***p < 0.001 compared to untreated controls; # *p* < 0.05 compared to cells treated with dexamethasone

To identify underlying molecular mechanisms of caffeine-induced suppression of CTGF, we analyzed mRNA expression of TGF-β1-3 in H441 cells following treatment with either dexamethasone or caffeine or a combination of both. Neither dexamethasone nor caffeine alone, nor a combination of both had any effect on the expression of TGF-β1 mRNA (Fig. 3a). TGF-β2 mRNA was slightly upregulated by dexamethasone (1.5 ± 0.3-fold, *p* = 0.0366; Fig. 3b), while there was no induction observable if caffeine was present. In contrast, both dexamethasone and caffeine significantly induced TGF-β3 mRNA either alone (1.8 ± 0.3-fold, *p* = 0.0066 and 2.4 ± 0.7-fold, *p* < 0.0001, respectively) or in combination (2.4 ± 0.6-fold, *p* = 0.0004; Fig. 3c). Induction of TGF-β3 mRNA was significantly higher in caffeine-treated cells in comparison to dexamethasone-treated cells (1.4 ± 0.4-fold, *p* = 0.0373; Fig. 3c). The given results may demonstrate that dexamethasone does not induce TGF-β1 but slightly induces TGF-β2 mRNA expression in H441 cells and that the inhibitory effects of caffeine could be, at least in part, mediated via a reduction of TGF-β2 but not TGF-β1 mRNA.

### Impact of cAMP, forskolin, cilomilast, and ruthenium red on CTGF mRNA expression in H441 cells

**Fig. 4:**
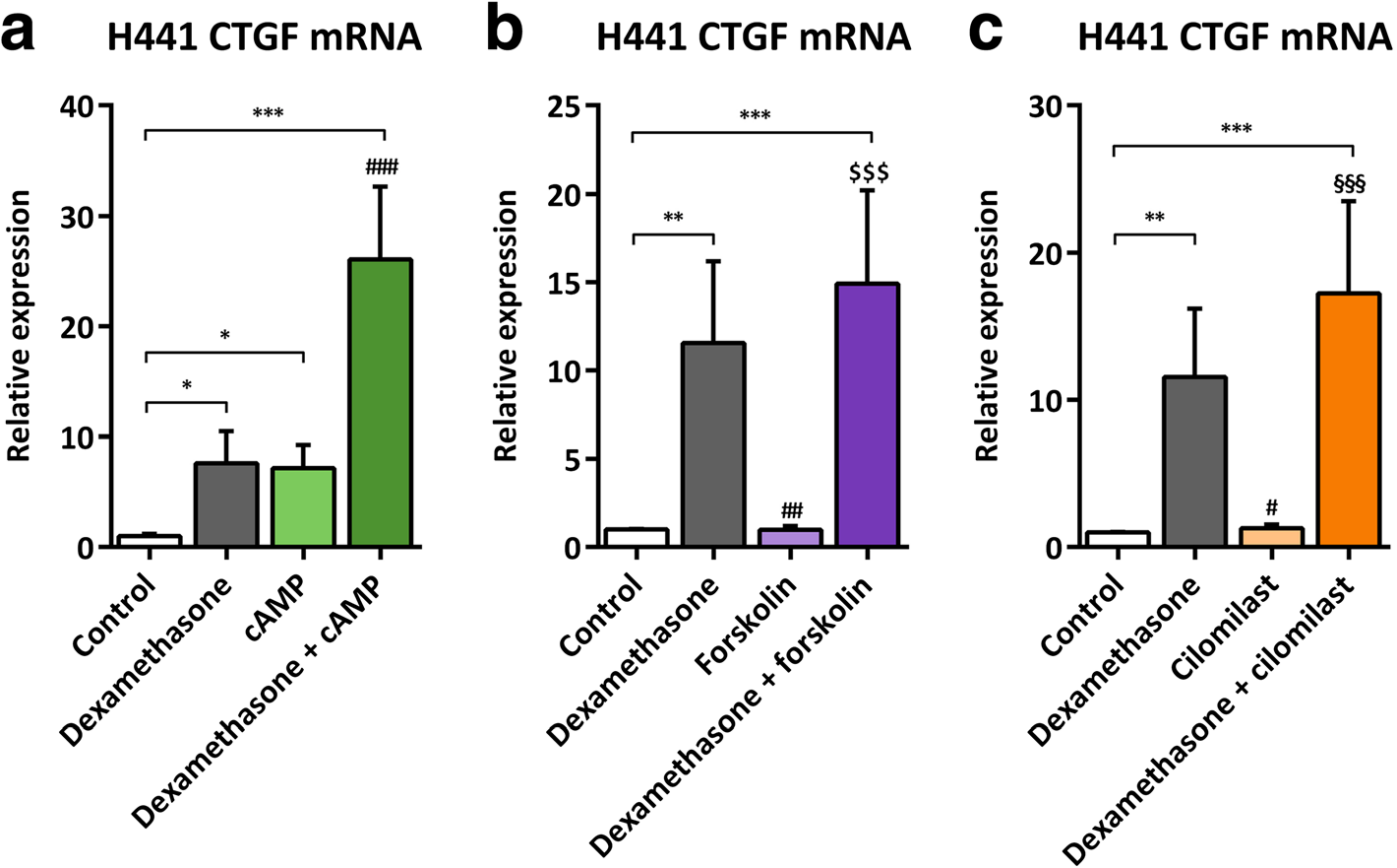
Impact of cAMP, forskolin, and cilomilast on CTGF mRNA expression in H441 cells. H441 cells were treated with 1 μM dexamethasone and/or 10 mM caffeine as well as 1 mM cAMP (**a**), 10 μM forskolin (**b**), and 10 μM cilomilast (**c**) for 24 h. qPCR against CTGF mRNA was performed as described in Methods. CTGF mRNA levels of H441 cells were normalized to GAPDH, and fold differences compared to untreated cells were calculated. Means + SD of n ≥ 2 independent experiments are shown. *p < 0.05, **p < 0.01, and ***p < 0.001 compared to untreated controls; # *p* < 0.05, ## *p* < 0.01, and ### *p* < 0.001 compared to cells treated with dexamethasone; $$$ *p* < 0.001 compared to cells treated with forskolin; §§§ *p* < 0.001 compared to cells treated with cilomilast

To reveal if a caffeine-induced inhibition of PDEs [52] was responsible for the observed inhibitory effect of caffeine, we treated H441 cells with dexamethasone and/or cAMP, the adenylyl cyclase activator forskolin, and the selective PDE-4 inhibitor cilomilast and subsequently measured CTGF mRNA levels. Unexpectedly, treatment of H441 cells with cAMP significantly increased CTGF mRNA expression (7.2 ± 2.1-fold, *p* = 0.0252) comparable to CTGF levels reached by dexamethasone (Fig. 4a). The combination of dexamethasone and cAMP further increased CTGF mRNA expression (26.1 ± 6.6-fold, *p* < 0.0001; Fig. 4a). Both forskolin (Fig. 4b) and cilomilast (Fig. 4c) did not affect basal or dexamethasone-induced CTGF mRNA levels, and, thus, were not able to antagonize the stimulatory effect of dexamethasone on CTGF expression. These results provide strong evidence that the inhibitory effect of caffeine on glucocorticoid-induced CTGF expression is not mediated via inhibition of PDEs. Since caffeine might activate ryanodine receptors potentially present on H441 cells to release Ca^2+^, we additionally performed experiments with the ryanodine receptor 1–3 antagonist ruthenium red to inhibit an activation by caffeine. However, we did not observe any modification of the inhibitory effect of caffeine when adding ruthenium red (data not shown).

### Impact of dexamethasone and caffeine on TNF-α mRNA expression and of TNF-α on CTGF mRNA expression in H441 cells

**Fig. 5:**
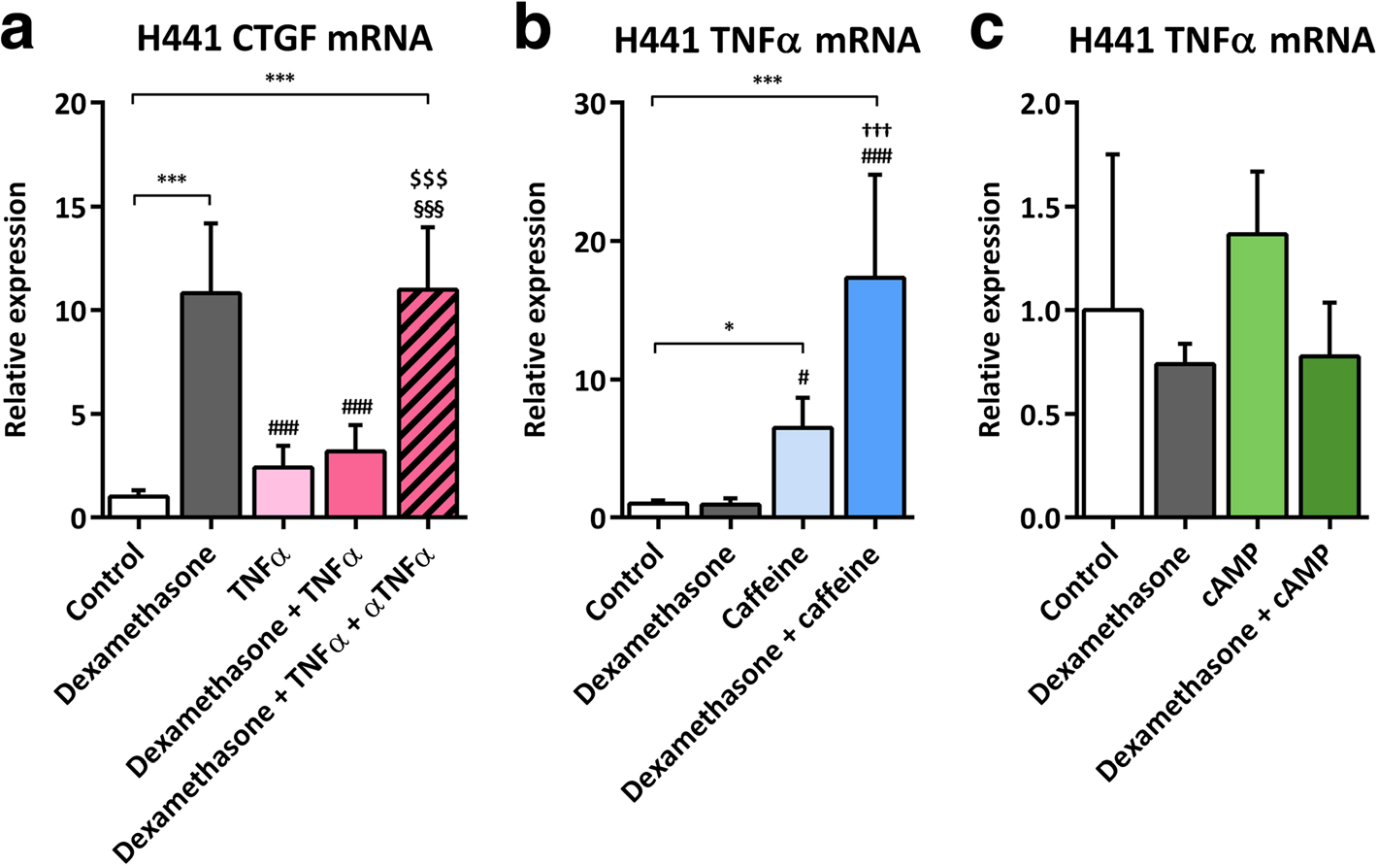
Impact of TNF-α, dexamethasone, caffeine, and cAMP on H441 CTGF and TNF-α mRNA expression. H441 cells were treated with 1 μM dexamethasone, 10 mM caffeine, 1 mM cAMP, 10 ng/mL recombinant TNF-α, and/or 5 μg/mL anti-human TNF-α antibody for 24 h. qPCR against TNF-α and CTGF mRNA were performed as described in Methods. CTGF (**a**) and TNF-α (**b**, **c**) mRNA levels of H441 cells were normalized to GAPDH, and fold differences compared to untreated cells were calculated. Means + SD of n ≥ 3 independent experiments are shown. αTNF-α, anti-TNF-α antibody; *p < 0.05 and ***p < 0.001 compared to untreated controls; # *p* < 0.05 and ### *p* < 0.001 compared to cells treated with dexamethasone; $$$ *p* < 0.001 compared to cells treated with TNF-α; §§§ *p* < 0.001 compared to cells treated with TNF-α and dexamethasone; ††† *p* < 0.001 compared to cells treated with caffeine

Since TNF-α has been shown to inhibit CTGF mRNA expression [56], we treated H441 cells with dexamethasone, caffeine as well as additional human TNF-α and/or anti-TNF-α antibody (Fig. 5a). TNF-α significantly reduced dexamethasone-induced CTGF mRNA levels (*p* < 0.0001). This effect was antagonized by the addition of anti-TNF-α antibody (*p* = 0.0001; Fig. 5a). Therefore, we speculated that an induction of TNF-α might be responsible for the observed suppressive effect of caffeine on CTGF mRNA expression. We analyzed TNF-α mRNA levels in H441 cells treated with dexamethasone alone or in combination with caffeine. Notably, suppression of CTGF mRNA induction in H441 cells was inversely correlated with induced expression of TNF-α mRNA upon treatment with caffeine alone (6.5 ± 2.2-fold, *p* = 0.0224) or in combination with dexamethasone (17.3 ± 7.5-fold, *p* < 0.0001; Fig. 5b). In accordance with these results, no induction of TNF-α mRNA was observed after treatment with cAMP or its combination with dexamethasone (Fig. 5c), indicating cAMP-independent suppression of CTGF expression and induction of TNF-α by caffeine. Thus, caffeine might antagonize glucocorticoid-induced expression of CTGF via cAMP-independent induction of TNF-α expression.

### Caffeine modulates the timely progression of dexamethasone-induced CTGF mRNA expression in H441 cells

**Fig. 6:**
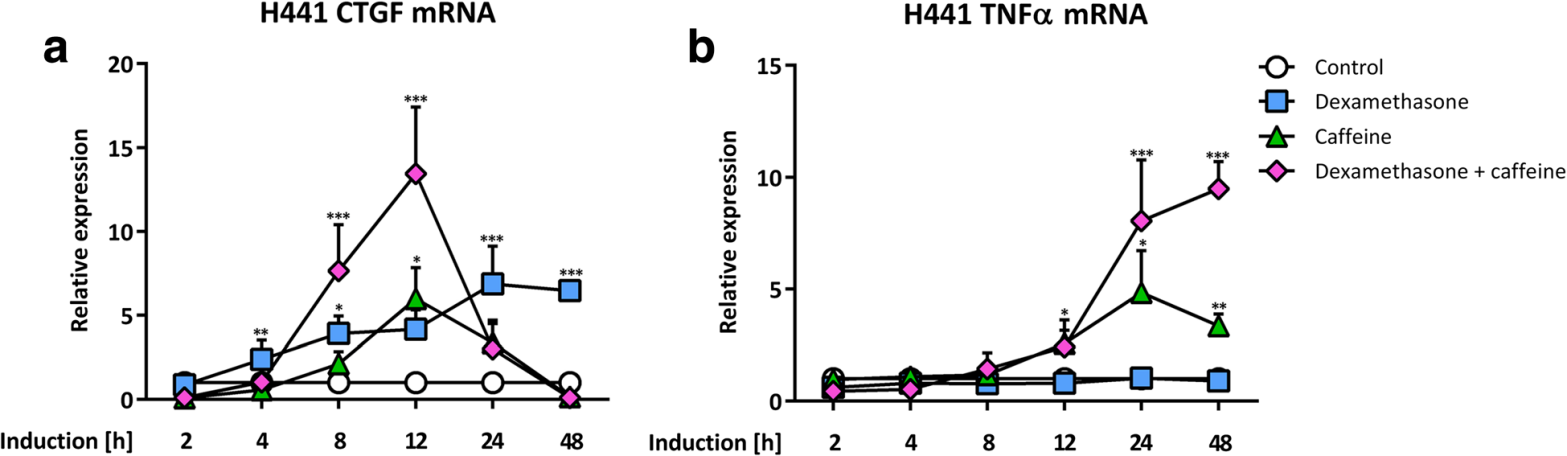
Treatment with caffeine modulates timely progression of dexamethasone-induced CTGF mRNA expression in H441 cells. H441 cells were treated with 1 μM dexamethasone and/or 10 mM caffeine for different time points as indicated. CTGF (**a**) and TNF-α (**b**) mRNA levels of H441 cells were normalized to GAPDH, and fold differences compared to untreated cells were calculated for each time point. Means + SD of n = 4 experiments are shown. *p < 0.05, **p < 0.01, and ***p < 0.001 increased mRNA expression compared to untreated controls

To reveal, if the observed reduction of glucocorticoid-mediated CTGF mRNA expression was timely restricted, we treated H441 cells with dexamethasone and/or caffeine for various time points. Stimulation with 1 μM dexamethasone resulted in significantly induced CTGF mRNA expression as early as 4 h, slowly further increasing until 24 h, and still significant after 48 h (6.5 ± 0.3-fold, *p* < 0.0001; Fig. 6a). Of note, contrary to our results at 24 h, caffeine induced CTGF mRNA expression at 12 h incubation in comparison to untreated cells (6.0 ± 1.9-fold, *p* = 0.0262). Simultaneously, parallel treatment with caffeine and dexamethasone led to a significant induction of CTGF mRNA at 8 h (7.7 ± 2.8-fold, *p* < 0.0001) and 12 h (13.5 ± 4.0-fold, *p* < 0.0001), being more pronounced than with dexamethasone alone. This induction of CTGF mRNA after the combined treatment was completely reversed at 24 h and 48 h. In contrast, after 48 h, levels of CTGF mRNA in cells treated with caffeine and dexamethasone were even lower than in untreated cells (0.11 ± 0.02-fold, *p* = 0.0002). In accordance to the absent CTGF mRNA induction upon co-stimulation with dexamethasone and caffeine, TNF-α mRNA expression (Fig. 6b) was significantly induced by caffeine not before 12 h (2.6 ± 1.0-fold, *p* = 0.0176), 24 h (4.8 ± 1.9-fold, *p* = 0.0181) and 48 h (3.4 ± 0.5-fold, *p* = 0.0031). This induction was even more pronounced at 24 h and 48 h after the combined treatment of caffeine with dexamethasone (8.1 ± 2.7-fold, *p* = 0.0002 and 9.5 ± 1.2-fold, *p* < 0.0001, respectively).

## Discussion

Our data reveal increased expression of CTGF in different lung cell lines by glucocorticoids. Our findings are in accordance with previous studies documenting glucocorticoid-induced expression of CTGF in various cell types [57–63]. If glucocorticoids may have adverse effects on airway remodeling processes via modification of pulmonary CTGF expression in vivo has yet to be defined. Although a short-term benefit of glucocorticoid treatment in severe forms of BPD has been sufficiently documented [64], concerns regarding significant unfavorable effects, including adverse neurological outcome, remain [65, 66]. Our data may add additional concerns in terms of glucocorticoid-related long-term impairments [34].

We observed induction of CTGF by different glucocorticoids on the transcriptional level in lung epithelial cell and fetal lung fibroblast models as well as on the translational level in the latter. Notably, these findings were not restricted to dexamethasone and betamethasone but were also confirmed for budesonide, prednisolone and hydrocortisone, often being considered to cause less side effects regarding treatment strategies in BPD [33, 67–70].

To the best of our knowledge, this is the first study to describe a glucocorticoid-induced expression of CTGF in human lung derived epithelial cells as well as fetal lung fibroblasts. These findings may be of considerable relevance, pointing to potential pro-fibrotic, CTGF-related adverse effects of glucocorticoids on lung development and tissue remodeling.

It has been suggested that inhibition of CTGF can prevent and reverse the process of fibrosis [26]. Combining pharmacological prophylaxis or treatment of CTGF-related pro-fibrotic lung injury with current therapies for inflammatory lung diseases may overcome current limitations of the latter [12]. In our setting, glucocorticoid-induced mRNA and protein expression of CTGF was attenuated by simultaneous exposure of lung epithelial cells and fetal lung fibroblasts to caffeine for 24 h and 48 h, respectively. Caffeine, already frequently used to reduce apnea of prematurity [40], has been ascribed to preventive effects on the development of BPD [71, 72]. In accordance to these clinical observations, our data point to the ability of caffeine to significantly suppress long-term glucocorticoid-induced CTGF expression on the transcriptional and translational level by modifying its timely progression. Moreover, caffeine may even reduce basal expression levels of CTGF.

Since highest levels of CTGF mRNA-induction by dexamethasone were detected in H441 cells, we focused on this cell line to identify the underlying molecular mechanisms of the stimulatory ability of glucocorticoids and the reductive ability of caffeine. We observed an induction of TGF-β2 and TGF-β3 mRNA by dexamethasone rather than of TGF-β1. Although TGF-β1 has been considered to be the predominant and most potent isoform in terms of CTGF induction [29], we hypothesize that glucocorticoid-induced expression of CTGF is likely to be independent of TGF-β1 in lung epithelial cells. In accordance to our data, TGF-β1-independent induction of CTGF expression has also been observed in cultured mouse fibroblasts as well as murine heart, kidney, and skin tissue [57]. The slight induction of TGF-β2 mRNA was no longer present after the addition of caffeine, indicating that TGF-β2 might be, at least in part, involved in the observed glucocorticoid-mediated induction of CTGF. Surprisingly, the induction of TGF-β3 was also observed for the treatment with caffeine to even higher amounts than by dexamethasone, indicating that alternative pathways are involved in the induction of CTGF. Although an induction of CTGF gene expression has also been described for TGF-β3 in fibroblasts [73], this TGF-β isoform has been ascribed to more positive effects in terms of pulmonary fibrosis than TGF-β1 [74] and even anti-scarring abilities have been assumed for TGF-β3 [75]. The observed induction of TGF-β3 mRNA might therefore point to a more anti-fibrotic ability of caffeine counteracting TGF-β1’s pro-fibrotic role.

Analyzing potential underlying mechanisms, we found the reduction of glucocorticoid-mediated CTGF expression by caffeine independent of PDE-inhibition, since treatment with cAMP or the adenylyl cyclase activator forskolin and the PDE4-specific inhibitor cilomilast showed no inhibitory effects. These results differ from those published for fibroblasts showing downregulation of TGF-β1-mediated CTGF expression via increased cAMP levels [76]. Accordingly, neither TGF-β1 and TGF-β3 signaling, nor the accumulation of cAMP seems to be the molecular basis for the inhibition of the glucocorticoid-mediated induction of CTGF by caffeine reported here. Although caffeine is able to increase the sensitivity of ryanodine receptors to cytosolic Ca^2+^ of airway smooth muscle cells [77], our experiments indicate that Ca^2+^ release by caffeine-mediated activation of ryanodine receptors is most likely not involved in the observed modifications of CTGF mRNA expression in lung epithelial cells. A further potential mechanism of caffeine is to act on bitter taste receptors, which have recently been identified in bronchial epithelial cells [78]. If these receptors are also expressed on lung epithelial cells or fibroblasts and may interact with the CTGF/TGF-β network has to be further elucidated.

Another possibility of caffeine’s mechanism of action regarding the observed downregulation of CTGF expression could be a potential impact on TNF-α which has been described as an inhibitor of CTGF expression [56]. Although TNF-α is a prototypic pro-inflammatory cytokine, its pleiotropic effects may often lead to opposing outcomes during the development of immune-mediated diseases [79]. We observed a significant increase of TNF-α mRNA by exposure of lung epithelial cells to caffeine and an inhibition of dexamethasone-induced CTGF expression by exogenous TNF-α. This is in contrast to studies reporting a reduction of TNF-α expression via nonselective PDE inhibition in monocytes, lymphocytes, and whole blood at lower caffeine concentrations [80–82]. The promoter of TNF-α contains a cAMP response element [83] and we found an induction of CTGF mRNA by cAMP which could have subsequently been provoked via caffeine-mediated increase of cAMP [53]. Thus, one may speculate that caffeine might have also induced TNF-α mRNA expression via cAMP in our setting, although we still observed unmodified TNF-α mRNA levels after exogenous cAMP, which is again questioning this assumption.

We may further attribute the caffeine-mediated induction of CTGF expression, observed at very early time points, to caffeine-mediated increases in cytosolic cAMP [53]. Moreover, an indirect induction of short-lived CTGF mRNA-degrading proteins by caffeine in consequence of this initial induction could be jointly responsible for the long-term reduction of CTGF. Such short-lived proteins have been suggested to be induced after upregulation of CTGF mRNA to prevent uncontrolled CTGF expression [14]. As far as cAMP is concerned, it is possible that its early rising levels provoked by caffeine [53] mediated an induction of CTGF mRNA which may be later antagonized by TNF-α.

There are some limitations of this study to be considered. Although CTGF may play a crucial role in development of lung fibrosis, it is likely to represent only one fraction of a much bigger network of regulating factors inducing fibrotic diseases which also need to be examined. Moreover, caffeine-induced suppression of CTGF expression was only observed at high in vitro concentrations of caffeine, possibly not reflecting physiologic conditions. Future studies will include investigations of primary cells to gain further insights into potential pro- and anti-fibrotic features of glucocorticoids and methylxanthines and the impact of co-medication. Concerning potential pro-fibrotic effects of glucocorticoids, their long-term usage should be carefully considered especially in preterm neonates. However, adverse pro-fibrotic effects may be attenuated by simultaneous and long-term administration of caffeine.

## Conclusions

To the best of our knowledge, this is the first study to describe a glucocorticoid-induced expression of CTGF in human lung derived cells. Our data may be of considerable relevance as they point to potential pro-fibrotic, CTGF-related adverse effects of glucocorticoids on lung development and tissue remodeling. Although attenuating preterm lung inflammation in the short run, application of glucocorticoids might negatively affect airway remodeling via induction of CTGF in the long term. The underlying mechanisms seem to be independent of TGF-β1. Thus, glucocorticoid treatment may be carefully considered in chronic inflammatory lung diseases, such as BPD in preterm infants. According to our data, a co-medication with caffeine may abrogate long-term glucocorticoid-induced CTGF expression and, therefore, help to attenuate the progression of BPD.

## Abbreviations

BPD: Bronchopulmonary dysplasia
cAMP: Cyclic adenosine monophosphate
CCN2: CCN family protein 2
CTGF: Connective tissue growth factor
GAPDH: Glyceraldehyde-3-phosphate dehydrogenase
H441: NCI-H441
PDE: Phosphodiesterase
TGF-β: Transforming growth factor
TNF-α: Tumor necrosis factor alpha
